# Evaluation of ELISA-Based Multiplex Peptides for the Detection of Human Serum Antibodies Induced by Zika Virus Infection across Various Countries

**DOI:** 10.3390/v13071319

**Published:** 2021-07-08

**Authors:** Maria del Pilar Martinez Viedma, Stephen Panossian, Kennedy Gifford, Kimberly García, Isis Figueroa, Leda Parham, Laise de Moraes, Lillian Nunes Gomes, Tamara García-Salum, Cecilia Perret, Daniela Weiskopf, Gene S. Tan, Antônio Augusto Silva, Viviane Boaventura, Guillermo M. Ruiz-Palacios, Alessandro Sette, Aruna Dharshan De Silva, Rafael A. Medina, Ivette Lorenzana, Kevan M. Akrami, Ricardo Khouri, Daniel Olson, Brett E. Pickett

**Affiliations:** 1 J. Craig Venter Institute, La Jolla, CA 92137, USA; mpmviedma@gmail.com (M.d.P.M.V.); gtan@jcvi.org (G.S.T.); 2 J. Craig Venter Institute, Rockville, MD 20850, USA; spanossian@student.umgc.edu; 3 Department of Microbiology and Molecular Biology, Brigham Young University, Provo, UT 84602, USA; kennedylincoln@icloud.com; 4 Instituto de Investigacion en Microbiologia, Universidad Nacional Autónoma de Honduras, Tegucigalpa, Honduras; kimfa_2010@hotmail.com (K.G.); isismaria_rodas@hotmail.com (I.F.); lparham29@hotmail.com (L.P.); ivettelorenzana@yahoo.com (I.L.); 5 Institute Goncalo Moniz, Fiocruz Bahia, Salvador 40296-710, Brazil; laisepaixao@live.com (L.d.M.); lilliangomes20@gmail.com (L.N.G.); vsboaventura@gmail.com (V.B.); 6 Departmento de Enfermedades Infecciosas e Inmunología Pediátrica, Escuela de Medicina, Pontificia Universidad Católica de Chile, Santiago H955+8Q, Chile; tcgarcia21@gmail.com (T.G.-S.); cperret@uc.cl (C.P.); rmedinai@uc.cl (R.A.M.); 7 Center for Infectious Disease and Vaccine Research, La Jolla Institute for Immunology, La Jolla, CA 92037, USA; dweiskopf@lji.org (D.W.); alex@lji.org (A.S.); dharshan_fom@kdu.ac.lk (A.D.D.S.); 8 Department of Medicine, Infectious Diseases Division, University of California San Diego, La Jolla, CA 92037, USA; 9 Hospital Universitário-Universidade Federal do Maranhão, São Luís 65000-000, Brazil; aamouradasilva@gmail.com; 10 Faculdade de Medicina da Bahia-Universidade Federal da Bahia, Salvador 40000-000, Brazil; kakrami@ucsd.edu (K.M.A.); ricardo.khouri@fiocruz.br (R.K.); 11 Instituto Nacional de Nutricion, Universidad Nacional Autonoma de Mexico, Mexico City 04510, Mexico; gmrps@unam.mx; 12 Department of Medicine, University of California San Diego, La Jolla, CA 92037, USA; 13 Genetech Research Institute, Colombo 00800, Sri Lanka; 14 Department of Paraclinical Sciences, Faculty of Medicine, General Sir John Kotelawala Defence University, Ratmalana 10390, Sri Lanka; 15 Department of Microbiology, Icahn School of Medicine at Mount Sinai, New York, NY 10029, USA; 16 Department of Pediatrics, Section of Infectious Diseases, University of Colorado School of Medicine, Aurora, CO 80045, USA; daniel.olson@childrenscolorado.org

**Keywords:** zika virus, antibody diagnostic, seropositivity, ELISA, virology, bioinformatics

## Abstract

Zika virus (ZIKV) is a mosquito-borne *Flavivirus* with a positive-sense RNA genome, which are generally transmitted through the bite of an infected *Aedes* mosquito. ZIKV infections could be associated with neurological sequelae that, and otherwise produces similar clinical symptoms as other co-circulating pathogens. Past infection with one member of the *Flavivirus* genus often induces cross-reactive antibodies against other flaviruses. These attributes complicate the ability to differentially diagnose ZIKV infection from other endemic mosquito-borne viruses, making it both a public health issue as well as a diagnostic challenge. We report the results from serological analyses using arbovirus-specific peptides on 339 samples that were previously collected from 6 countries. Overall, we found that our multiplexed peptide-based ELISA was highly efficient for identifying ZIKV antibodies as early as 2 weeks post infection, and that it correlates with microneutralization, plaque reduction neutralization tests (PRNTs) and commercial tests for ZIKV in previously characterized samples. We observed that seropositivity varied by patient cohort, reflecting the sampling period in relation to the 2015–2016 ZIKV outbreak. This work evaluates the accuracy, specificity, and sensitivity of our peptide-based ELISA method for detecting ZIKV antibodies from geographically diverse regions. These findings can contribute to ongoing serological methods development and can be adapted for use in future studies.

## 1. Introduction

Zika virus (ZIKV) is a mosquito-borne *Flavivirus* that contains a positive-sense, single-stranded RNA genome. The virus is closely related to other members of this family, which is not limited to dengue virus (DENV), Ilheus virus (ILHV), Japanese encephalitis virus (JEV), St. Louis encephalitis virus (SLEV), West Nile virus (WNV), and yellow fever virus (YFV). Although ZIKV is primarily spread through the bite of an infected *Aedes* mosquito, cases resulting from sexual transmission, vertical transmission, and blood transfusion have been reported [1,2,3,4]. ZIKV caused sporadic and isolated outbreaks in Africa, Southeast Asia and the Pacific Islands before early 2015 [5,6]. In 2015–2016, the virus rapidly spread throughout Latin America and the Caribbean, producing unprecedented case numbers. Most ZIKV infections are characterized as either asymptomatic or having only mild symptoms; however, the 2015–2016 epidemic demonstrated that ZIKV is a neuropathic virus that is associated with serious neurological abnormalities such as Guillain-Barre syndrome in adults and congenital Zika syndrome, which includes microcephaly in fetuses [7]. Due to the continued threat of ZIKV re-emergence around the world, there is still a pressing need for continued surveillance to rapidly identify the risk of emerging outbreaks.

Broad serological cross-reactivity between species within the *Flavivirus* genus using existing serological methods has been well established, but also represents a major barrier for the accurate assessment of disease prevalence rates [8,9,10,11,12]. In addition, the use of traditional whole viral antigen in enzyme-linked immunosorbent assays (ELISAs) poorly differentiates among infection with other flaviviruses (e.g., DENV) that co-circulate in the same regions via the same mosquito vector(s) [13]. Another confounding factor is the similar clinical presentation to other mosquito-borne diseases, such as chikungunya virus—a member of the *Alphavirus* genus of the *Togaviridae* family [14,15,16], which cross-reacts against other related taxa including Mayaro virus [17]. A limitation of prior ELISA-based diagnostic efforts targeting whole viral envelope (E) or nonstructural protein 1 (NS1) proteins is the consistent report of cross-reactive polyclonal serum resulting in false-positive results among a subset of mosquito-borne flaviviruses [18]. Separate studies have evaluated both serologic and genetic detection methods [19], generated ELISA reagents to detect ZIKV infection [20], produced a set of monoclonal antibody reagents that are capable of detecting virus ZIKV NS1 protein [21], anti-dengue NS1 antibodies with variable degrees of success [22].

We have previously predicted eight sets of 10 taxa-specific viral peptides that could be applied as differential serodiagnostics to detect prior infection with a panel of eight mosquito-borne viruses [23]. These peptides were shown to have greater than 98% specificity and sensitivity for the target taxon based on a statistical sequence analysis, were predicted to be exposed on the protein surface based on three-dimensional protein modeling and were predicted to contain B-cell epitopes. These predicted diagnostic peptides were subsequently multiplexed and evaluated for reactivity in well-characterized human sera using both a peptide array and an ELISA platform. The ELISA results from these experiments showed acceptable specificity and sensitivity values for ZIKV in well-characterized human sera [24].

The aim of the current study was to further quantify the performance of our previously reported multiplexed ZIKV diagnostic peptides in an ELISA format. The performance of this peptide-based ELISA method for detecting anti-ZIKV antibodies was evaluated against human sera from diverse countries (e.g., Brazil, Chile, Guatemala, Honduras, Mexico, and Sri Lanka) as well as at various timepoints during the 2015–2016 ZIKV epidemic. Furthermore, we aimed to better understand the ability of our peptide-based ELISA platform to detect seroconversion and seropositivity during the early and intermediate stages of the ZIKV epidemic.

## 2. Materials and Methods

### 2.1. Serum Sources

Appropriate ethical approval was obtained prior to sera collection. Three hundred seventy-nine serum samples were collected previously from individuals diagnosed with ZIKV or DENV in: Brazil (108), Chile (27), Guatemala (200), Honduras (32), Mexico (3), and Sri Lanka (9) at various timepoints during the ZIKV epidemic (Supplemental Table S1). A subset of these sera had been partially characterized with at least one standard or commercial serological assay.

For the Brazilian samples, informed written consent was obtained under protocol 1.657.324 and 2.824.637, which was reviewed and approved by the School of Medicine the Federal University of Bahia, Brazil, and under protocol 2.111.125, which was reviewed and approved by the University Hospital of the Federal University of Maranhão, Brazil, before the start of sample collection.

For the Chilean samples, informed written consent was obtained under protocol 16–066, which was reviewed and approved by the Scientific Ethics Committee at Pontificia Universidad Católica de Chile (PUC) before the start of sample collection. Collected samples consisted of sequential samples belonging to 9 patients with confirmed ZIKV or DENV infection, which were obtained longitudinally at one week, two weeks, or two months, while one sample was collected from a pregnant patient at five months post onset of symptoms or diagnosis.

The Guatemalan study was approved by the Colorado Multiple Institutional Review Board (COMIRB), the Universidad del Valle de Guatemala (UVG) Institutional Review Board, and the Guatemala Ministry of Health National Ethics Committee. Whole blood samples were collected in the field between Oct 2015 and Feb 2016, refrigerated immediately, and serum separated within 24 h then frozen at −20 °C. Samples were maintained on-site for up to 4 weeks then shipped on dry ice and subsequently stored at −80 °C until undergoing testing.

The Honduran samples consisted either of spent diagnostic samples or were collected under an IRB protocol (number 00003070) approved by the Scientific Investigation Unit at the Universidad Nacional Autónoma de Honduras Medical Science Faculty. Blood samples from routine care were obtained from the Hospital Escuela Universitario between June 2016 and June 2018. The cohort of patient ages ranged from 34 days to 73 years and consisted of the general population, pregnant women, and children with microcephaly.

The Mexican sample used in this study was a spent diagnostic biospecimen that was used to determine the ideal serum dilution. The samples from Sri Lanka were collected as part of a collaboration between the Genetech Research Institute, Colombo in Sri Lanka and the La Jolla Institute for Immunology. As such, this study was performed under an approved IRB protocol (VD-085) approved by the La Jolla Institute for Immunology IRB committee, with additional approvals from the University of Colombo, Ethics review committee, which served as the NIH approved IRB for the Sri Lanka site.

The control convalescent sera were obtained from three sources including BEI Resources (Catalog Numbers: NR-50226, NR-50231, NR-50896, NR-50900, and NR-50902), the World Reference Center for Emerging Viruses and Arboviruses reference collection, and the United States Centers for Disease Control and Prevention.

### 2.2. High-Throughput ELISA Plate Preparation, Quantification, and Normalization

A liquid-handling robot (BIOROBOT Rapid Plate, Zymark; Watertown, MA, USA) was used to ensure accuracy and reproducibility of the plate preparation process. Firstly, 2 ng of chemically synthesized peptides (LifeTein; Somerset, NJ, USA) were diluted in 50 μL of double-distilled H_2_O. Briefly, these synthetic peptides consisted of seven sets of eight to ten diagnostic peptides (Supplemental Table S2), which were previously predicted to be specific to each of the selected mosquito-borne virus taxa. Pairs of all peptides for each virus were then plated in duplicate wells until all 68 peptides were represented in 34 wells in duplicate (total of 68 experimental wells) on a 96-well Immulon 4HBX plate (ThermoFisher; Waltham, MA, USA, cat. # 3855). An established protocol, which was used to evaluate characterized human sera, was then followed to evaluate the performance of the peptides on other sets of human sera [24]. Human IgG protein (abcam; Waltham, MA, USA, cat. # ab91102), complement component C1q from human serum (sigma, cat. #: C1740), and labelled secondary antibody (ThermoFisher; Waltham, MA, USA, cat. #: A18817, RRID: AB_2535594) were used to coat control wells prior to incubation at 4 °C overnight. After overnight incubation, 100 μL of blocking buffer (PBS + 5% BSA) were added to each well prior to incubation for 2 h at room temperature (RT). Plates were then subjected to three washing steps with washing buffer (PBS + 0.05% Tween 20). Samples from the Mexican study were used to determine the ideal serum dilution. Separate assays evaluating dilutions of 1:25, 1:50, and 1:100 showed that diluting samples 1:25 was ideal. Consequently, human serum was diluted 1:25 in blocking buffer and 50 μL of this solution was added to each well before incubating for 2 h at RT. Each plate was washed four times with washing buffer prior to adding 50 μL of Horseradish peroxidase-conjugated anti-human IgG antibody (ThermoFisher; Waltham, MA, USA, cat. #: A18817, RRID: AB_2535594; 0.1 mg/mL diluted 1:20,000) to each well and then incubated at RT for 2 h. Plates were then washed four times before incubating at RT for 30 min with 75 μL of TMB substrate (abcam; Waltham, MA, USA, cat. # ab171523). Seventy-five μL of stop solution (abcam; Waltham, MA, USA, cat. # ab171529) was then added to each well and a BioTek-synergy HT plate reader was used to quantify the fluorescence in each well at 450 nm within 15 min [24]. Quantified absorbance values for each well of each ELISA plate were then normalized using an existing algorithm that incorporates fluorescence values of control wells to enable cross-plate comparisons [25]. A set of control samples were evaluated previously with an identical workflow [24].

### 2.3. Computational Processing of Peptide ELISA Data

Normalized absorbance values greater than 2.5, between 1.5 and 2.5, or less than 1.5 for duplicate wells on each plate were categorized as “positive”, “borderline”, or “negative” for the target virus as described previously [24]. Briefly, these numerical values consist of dividing background-corrected experimental wells by the background-corrected positive control wells. For ZIKV samples specifically, a “positive” result also involved minimizing potential cross-reactivity by ensuring that the observed normalized values in ZIKV wells was at least twice as large as the observed normalized values for any wells with a putative positive signal for DENV, which was based on the MAC-ELISA guidelines from the US Centers for Disease Control and Prevention. Samples that were categorized as “borderline” were combined with “positive” samples for this work.

In addition to these data processing steps, a set of quality control criteria were implemented that included (1) ignoring results from assays where high levels of background signal were detected in wells with no antigen, (2) disregarding results from assays where all tested viruses were detected in the same sample and was deemed as a potentially contaminated ELISA plate, and (3) ensuring that a background-normalized value of at least one was calculated for the secondary antibody control wells to identify plates with high levels of background signal and/or low signal in positive controls. The second quality control procedure was originally established to minimize the probability of a false positive result when multiple viruses were being evaluated simultaneously. The focus on ZIKV seropositivity similarly benefits from this procedure since it enables the identification of rare samples that are positive for all virus taxa, which is likely a laboratory artifact. Natural infection with all viral taxa represented on the plate in any given country is extremely unlikely since certain viruses represented on the ELISA plate were not endemic in each of the areas tested.

Dividing the samples into these categories, based on the quantified ELISA results, enabled the calculation of seroconversion rates in longitudinal characterized samples and seropositivity rates for individual uncharacterized clinical samples.

### 2.4. Validation Using Mikrogen Commercial Assay

The commercial Mikrogen serology test was performed, according to the manufacturer’s protocol, to validate a subset of samples using an independent diagnostic platform (recomLine Tropical Fever IgG, cat.# 7872). Briefly, this procedure involved incubating 20 μL of undiluted serum to the test strip and incubating for one hour with shaking. The test strips were then washed and incubated with the conjugate solution before washing and incubating with substrate solution. The reaction was then washed three times to stop the reaction and the test strips dried prior to interpretation.

### 2.5. Validation Using PRNT Assay

Plaque reduction neutralization tests (PRNTs) were performed on the Brazilian samples as previously reported [26], with minor modifications. The cutoff value for PRNT positivity was defined as 90% (PRNT90). PRNT90 was performed to determine the maximum serum dilution (ZIKV-1:8 to 1:1024) needed to reduce arbovirus plaque formation by 90% among Vero cells. For this, the ZIKV virus strain isolated in Brazil was used. All sera were heat-inactivated (56 °C, 30 min) prior to neutralization testing. The serum samples were diluted in serial dilutions method, using modified Dulbecco Eagle medium containing 2% fetal calf serum and 1% of Penicillin/Streptomycin as diluent. Next, virus suspension was mixed to each serum dilution and were incubated at 37 °C for 60 min. A final volume of each serum dilution and virus (50–100 ffu/well—12-well cell culture plate) mixture was transferred to a well containing Vero cells and then incubated at 37 °C for 60 min. Following incubation, 0.3% agarose solution was added, and plates were reincubated at 37 °C for 5 days. Reactions were then revealed using 2% naphthol blue black solution. Titers ≥ 10 (Lab 1) or ≥20 (Lab 2) were considered positive.

### 2.6. Validation Using Microneutralization Assay

Samples from Guatemala were subjected to ZIKV microneutralization assays following a previously established protocol [27]. Briefly, diluted sera were evaluated in 96-well format with two-fold serial dilutions allowed to neutralize 600 plaque-forming units of ZIKV strain PRVABC59 in each well for one hour at 37 °C. The 50% tissue culture infectious dose (TCID50) was determined on a separate titration plate by performing three-fold serial dilutions of 600 PFU in 50 µL across each row of a 96-well plate prior to incubation for 1 h at 37 °C. A fresh aliquot of 80,000 Vero cells was then added to each well prior to incubation for four days at 37 °C. The cells were then washed, fixed, blocked, and incubated with the HB112-4G2 pan-flavivirus monoclonal antibody prior to calculating the end point titer by determining the dilution that neutralized 50% of the signal.

## 3. Results

### 3.1. Peptide-Based ELISA vs. Mikrogen Commercial Assay

Our study focuses on comparing the performance of our multiplexed custom-designed peptide panel to detect antibody responses to mosquito-borne viral taxa including species within the *Flavivirus* genus and CHIKV with our peptide-based ELISA (Supplemental Table S3). Our ELISA results were evaluated against various validation assays that were performed on subsets of human sera (Table 1). To begin, we evaluated our peptide ELISA against the commercially available Mikrogen serological test kit. We focused this comparison on well-characterized control sera that had been evaluated previously [24]. These control sera consisted of samples with little cross-reactivity to ZIKV, reactivity to both ZIKV and DENV, only DENV reactivity, or no ZIKV reactivity, which were originally characterized using a mix of ZIKV MAC-ELISA, ZIKV PRNT, DENV PRNT, viral quantitative real-time PCR, and/or IgM reactivity to ZIKV.

Our initial analysis consisted of using the control sera to compare the calculated specificity and sensitivity values from six samples evaluated with our ELISA method against the Mikrogen test. This commercial test is qualitative and has 100% sensitivity and 95% specificity for detecting anti-ZIKV IgG. Comparing the results from the relatively low number of six samples that had results from both our custom peptide-based ELISA and the Mikrogen assay showed that our ELISA had high sensitivity and relatively low specificity for ZIKV (100% and 50%, respectively) (Supplemental Table S3).

We also observed that the sensitivity and specificity for CHIKV were unreliable (100% and 25%, respectively), while the sensitivity and specificity values for DENV were also unreliable (100% and 0%, respectively) for our ELISA test when compared against the Mikrogen test. These sensitivity and specificity values were impacted by the small samples size; however, the specificity value is similar to what was previously reported for this custom peptide platform. The normalized ELISA results for the control samples, together with the 339 experimental samples evaluated in this study are available for reuse (Supplemental Table S4).

### 3.2. Comparison of Peptide-Based ELISA against DENV qRT-PCR

We next examined a collection of nine samples from Sri Lankan patients that were collected shortly after the onset of symptoms and were previously characterized as qRT-PCR-positive and/or IgM-positive for DENV (Supplemental Table S5). There was some concordance (~70%) with the prior clinical IgM serological tests for these samples, which could have partially been due to cross-reactivity to antibodies from past infection(s). Overall, these observations validate our findings from the control samples and suggest that additional work should be performed to optimize the performance of our selected peptides against some of the dengue viruses.

### 3.3. Peptide-Based ELISA vs. IgG in Longitudinal Samples from Chilean Patients

We next wanted to better understand the ability of our multiplexed peptide-based ELISA to detect seroconversion in clinical samples that had been characterized as positive for ZIKV during acute infection. As such, we examined the peptide ELISA results from 19 longitudinal Chilean samples that were collected from seven patients (Supplemental Table S6). These patients were clinically diagnosed with PCR-based tests for infection with ZIKV one to two weeks after initial presentation at the clinic. IgG-based serological tests were performed for samples collected after two or more months. Evaluating these samples with our assay was extremely valuable since ZIKV is not endemic in Chile. Rather these samples were collected from travel-associated cases.

These experiments yielded sufficient data for at least two timepoints for five of the seven ZIKV patients (Table 2). Patient number six had more than one sample collected at least one week after diagnosis at the clinic that were subsequently tested with our peptide-based ELISA. Patient number five had a single sample taken at 5 months post-infection, but the results did not meet our QC criteria and were excluded. Similarly, none of the three longitudinal samples from patient number one passed our QC metrics and were also excluded. We found evidence that four of the five ZIKV patients seroconverted within weeks after onset of symptoms/diagnosis. Interestingly, ZIKV patient number six was found to be asymptomatic when a sample was collected and tested at the clinic. ZIKV patient number seven did not display evidence of seroconversion two months after infection either with clinical testing or with the peptide-based ELISA, and was consequently categorized as a false-positive result. We also found evidence of IgG seroconversion occurring approximately two weeks after diagnosis and/or onset of symptoms in three of the five valid samples. These data indicate that our peptide-based ELISA allowed us to accurately detect ZIKV-specific antibodies within two to three weeks after the onset of symptoms in the absence of other cross-reacting flaviviruses. This suggests that our ZIKV-specific peptides could detect IgG antibodies within two weeks after the onset of symptoms outside of pregnancy.

### 3.4. Peptide-Based ELISA vs. qRT-PCR and PRNT Assays in Characterized Brazilian Serum Samples

We next used a cohort of Brazilian patients suspected of arbovirus infection to test the ability of our multiplexed peptide-based ELISA to identify seropositive samples for ZIKV (Supplemental Table S7). The seven ZIKV-positive biospecimens collected from these patients had previously been extensively characterized with at least one RNA-based method, with a subset giving positive results with IgG-based methods. These characterization assays included RT-qPCR of plasma, urine, oral swab, and whole blood during acute infection or plaque reduction neutralization tests (PRNTs). To better quantify the performance of our peptides against other commonly used assays we compared the results from our peptide-based ELISA results against those from the PRNT assay. PRNTs are a well-accepted low-throughput assay to quantify the ability of serum to prevent the virus from entering cells with extremely high sensitivity and specificity. Quantitative PRNT results were only available for six of the seven putative ZIKV positive samples (Table 3), which affected the statistical power of this comparison. Even so, we observed six of the seven positive PRNT assays gave positive results with our peptide-based ELISA. Interestingly, our assay also detected anti-ZIKV IgG antibodies in the seventh patient who had positive RT-qPCR results in both urine and blood. Overall, this comparison shows that our assay has ~85% concordance with the PRNT results, which is similar to the values that were previously obtained while testing characterized sera with our assay.

### 3.5. Peptide-Based ELISA to Estimate Seropositivity among Individuals in ZIKV-Endemic Regions

After validating our multiplexed peptide-based ELISA on multiple characterized samples, we next wanted to use our tool to quantify the ZIKV seropositivity rate in clinical samples collected from additional geographic regions, including Brazil (*n* = 36), Guatemala (*n* = 200), and Honduras (*n* = 33) at the early and intermediate stages of the ZIKV epidemic. This involved testing these 269 clinical samples (Supplemental Table S8), that passed our QC procedure.

**Figure 1:**
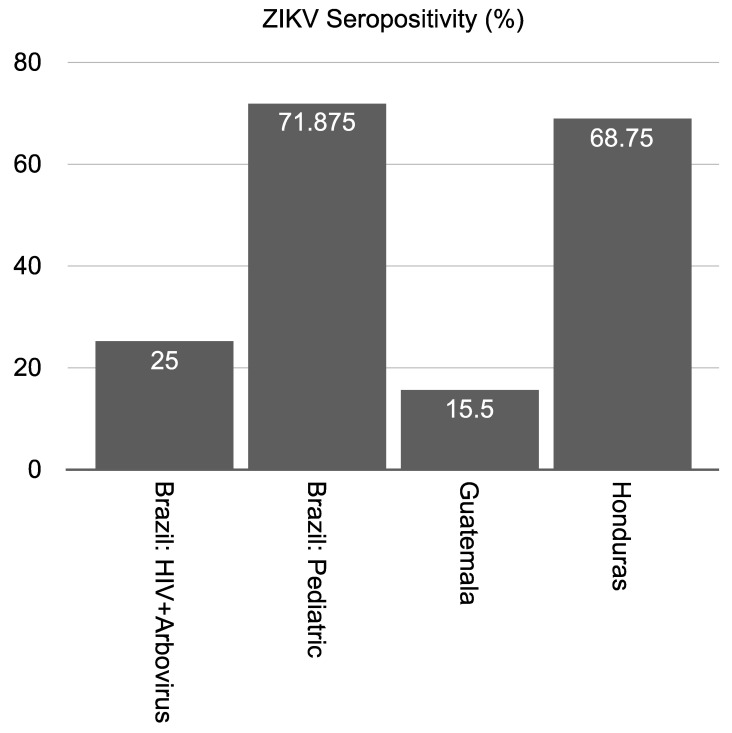
ZIKV seropositivity quantified across each uncharacterized patient cohort at the early and intermediate stages of the ZIKV epidemic. Positivity rate was measured according to the results from the peptide-based ELISA method on human samples collected from four countries with endemic circulation of ZIKV.

The quantified seropositivity results vastly differed by country and patient cohort (Figure 1). We calculated the range of seropositivity for each country and cohort metadata based on the same ELISA confidence categories that were described above. Doing so revealed that the percentage of people who were seropositive as a result of previous infection with ZIKV were 69%, 16%, and 69% for Brazil, Guatemala, and Honduras, respectively.

After looking at the seropositivity percentages for sera collected in each country, we analyzed each cohort further to identify any underlying biological trends. Specifically, we evaluated two Brazilian cohorts that consisted of either a small number of HIV-infected patients, or a pediatric cohort with IgM data for ZIKV in a subset of patients.

The first set of sera from Brazil consisted of four HIV-infected adults collected in August or September of 2018. These samples were collected due to patients displaying signs and symptoms of arboviral disease, but they had not been characterized for ZIKV previously. One of these samples was seropositive as quantified with our assay and had a HIV viral load below the limit of detection of qRT-PCR. Two of these patients were seronegative for ZIKV with our peptide-based ELISA. One of these patients had a HIV viral load of 79 copies per milliliter on the date of sample collection, while the second had no detectable viral load. Conversely, one sample had high levels of background on our ZIKV assay and undetectable HIV viral load (Table 4).

The second set of Brazilian biosamples was collected from pediatric cases of Zika microcephaly syndrome in patients between 16 and 35 months of age in Maranhão (Table 5). These patients had previously undergone IgM- and IgG-based ELISA assays as well as computed tomography (CT) scans in utero. Specifically, all 17 samples that tested positive by both CT and a separate IgG ELISA assay also tested positive with our peptide-based ELISA. Additionally, all five samples that were shown to be positive with separate ELISA assays measuring IgM were categorized as seropositive for ZIKV with our assay. Overall, our peptide-based ELISA method measured the seropositivity rate of 72% in this population. Although we cannot determine whether our assay detects IgG from the fetus/newborn or from the mother, these results suggest that our assay could be used to confirm the presence of anti-ZIKV IgG antibodies during or shortly after gestation.

### 3.6. Comparison of Peptide-Based ELISA with Microneutralization Assays in Uncharacterized Guatemalan Samples

We performed microneutralization (MN) assays on 175 samples collected from Guatemala to independently validate the accuracy of our multiplexed ELISA approach. The goal of these experiments was to quantify the performance of our ELISA antibody titer results with those from the microneutralization assays. This would also enable us to compare the relatively low antibody titers seen in the ELISA against the neutralization levels observed in these samples collected from Guatemala. Overall, we found that the specificity of our peptide-based ELISA, compared to the MN to be moderate while the sensitivity was extremely low (Table 6, Supplemental Table S9). While the specificity for these samples is somewhat lower than when compared against the PRNT data reported previously, it still is similar to what was reported for the well-characterized control sera above [24].

## 4. Discussion

Here, we show that our multiplexed custom-designed peptide panel was able to determine seropositivity against ZIKV infection in previously collected blood samples from human populations across multiple countries. This seropositivity study expanded on our prior work that identified a peptide pool specific for detecting ZIKV specific antibodies that enabled us to differentially diagnose against related members of the *Flavivirus* genus. The ELISA reagents evaluated here represent a valuable complement to others that have been reported previously [28] and generally provided concordant ZIKV seropositivity results in samples that had been characterized with molecular and/or serological methods.

The ability of our peptide-based ELISA to accurately replicate previous ZIKV serological test results in characterized Chilean and Brazilian samples increases the relevance of these diagnostic tools. Our assay enabled us to discriminate true seroconversion from background in one patient and the detection of anti-ZIKV IgG antibodies less than 2 weeks post-diagnosis in a subset of the Chilean samples. To our knowledge, this finding has only been reported in a single study that showed the detection of anti-ZIKV antibodies at 13 days post-infection in a cohort of pregnant women [29]. This provides an improvement when compared with many IgG-based assays, which are only capable of discriminating antibody presence approximately one month after infection [30,31,32].

The number of samples evaluated from each country in this study, the time of collection relative to the onset of the outbreak, and the bias towards evaluating samples from patients who presented with symptoms at the clinic may contribute to the observed differences in seropositivity, specificity, and sensitivity between countries in this study. It is therefore possible that results from future studies with our assay would be affected if the human population in each country was sampled in a more random and a representative way. Our custom peptide-based ELISA produced a consistent range of qualitative results when compared against the ensemble of established assays used to evaluate its performance. This observation further increases our confidence in the performance of our peptide-based ELISA assay.

Our multiplexed peptide approach differs from many of the existing methods that require separate assays to be performed to detect a single virus [32,33,34,35]. This approach addresses a pressing need since rapid detection of antibodies to these common, endemic, and co-circulating mosquito-borne viruses during the early stages of an outbreak may facilitate public health surveillance efforts. A rapid and accurate ZIKV diagnostic method would also improve serological detection of at-risk populations and to conduct large serological studies to identify potential outbreaks due to increases in seroprevalence in the population. As expected, comparing the performance, including sensitivity and specificity, of ELISA-based platforms against other diagnostic methods has shown varying levels of performance [36].

Of note, there are also potential limitations of the peptide-based ELISA approach employed in the current study. First, while applying the quality control metrics dramatically improved the serological sensitivity of our peptides, the process may introduce bias into the results generated from the uncharacterized sera. Second, seroconversion assumes that the vast majority of samples collected longitudinally from infected people show evidence of an IgG response shortly after infection. Additional studies are needed to determine whether asymptomatic ZIKV infections consistently generate a similar antibody profile to what is observed after symptomatic infections. Immunological imprinting may also play a role in the person-to-person serological diversity observed in the adaptive immune response [37,38]. Third, although our peptides were designed to identify antibodies across an array of flaviviruses and CHIKV, the specificity and sensitivity of these reagents limited the major focus of the current study only to ZIKV. Although the samples included in this study were characterized with various methods, the consistent specificity of our peptides supports further research into optimizing this peptide-based ELISA approach for serological testing.

The differing performance of our peptides in patient samples collected from multiple countries is also important. For example, the pediatric samples from Brazil showed good overall performance, while the adult samples from Guatemala showed lower performance. This observation suggests that our peptides perform better in sera where cross-reactivity is less common. These findings also support known inconsistencies that exist between various molecular and serological methods. Future studies could focus on adjusting and optimizing the peptides as well as their combination and arrangement on the plate to improve the performance of this peptide-based assay.

Secondary analysis of these data and/or meta-analysis of multiple seropositivity datasets could augment our knowledge of the history of infection for flaviviruses. It could also be useful to perform follow-up experiments using the same peptide-based ELISA method in the future to determine how long antibodies circulate in people after the initial virus exposure, or whether these peptides are capable of detecting the human anti-ZIKV IgM antibody response.

## 5. Conclusions

In summary, the serological ELISA results for the 339 characterized and uncharacterized samples that were evaluated in this study show that our method has lower-than-expected sensitivity with moderate specificity across diverse serological and genetic backgrounds when compared to existing methods. These results improve our understanding of the use of peptides to identify specific antibodies against ZIKV in uncharacterized samples from various countries obtained during the 2015–2016 epidemic in the western hemisphere. In addition, the possibility of detecting ZIKV-specific IgG antibodies prior to two weeks after diagnosis with our assay validates similar findings in pregnant women. These findings are relevant to a variety of applications including work in serological surveillance, vaccine development, and characterizing the host immune response during and after ZIKV infection.

## Figures and Tables

**Table 1:** Sera collections, validations assays, and comparisons reported in this study.

		Collection							
		Control	Sri Lanka	Chile	Brazil (HIV)	Brazil (Pediatric)	Brazil (Arbovirus)	Honduras	Guatemala
	Number Starting Samples	6	9	27	108			32	200
	Number Samples (after QC)	6	9	19	4	22	32	32	175
Validation Assays	Mikrogen IgG	✓ *							
	qRT-PCR		✓ *	✓			✓ *		
	IgM (ELISA)		✓			✓	✓		
	IgG (ELISA)			✓ *		✓ *			✓
	Computed Tomography					✓ *			
	CDC MAC ELISA						✓		
	PRNT						Y *		
	Micro-neutralization								✓ *
	BOB-ELISA								✓
	Peptide ELISA	✓	✓	✓	✓	✓	✓	✓	✓

✓: Validation assays performed on samples from the specified patient cohort. *: Validation assays compared directly with peptide-based ELISA.

**Table 2:** Results from characterized Chilean samples using peptide-based ELISA.

Patient Number	Clinical Diagnosis	1 Week **	2 Weeks **	2 Months **	5 Months **	Notes
1	ZIKV	ND	ND	ND	*	
2	ZIKV	*	Neg	Pos	*	
3	ZIKV	*	Pos	Pos	*	
4	ZIKV	Pos	Pos	ND	*	
5	ZIKV	*	*	*	ND	
6	ZIKV	Neg,ND,Pos ^+^	Pos	Pos	*	Asymptomatic
7	ZIKV	ND	Pos	Neg	*	False positive

^+^ Multiple tests were performed on samples collected during week one. ND: not determined. Pos: positive result for target virus. Neg: negative for target virus. *: no sample collected. **: time is measured from the day the patient was first diagnosed at the clinic.

**Table 3:** Comparison of peptide-based ELISA to nucleic acid and serological assays.

Sample ID	qRT-PCR (Plasma)	qRT-PCR (Urine)	qRT-PCR (Oral)	qRT-PCR (Blood)	CDC MAC-ELISA	Euroimmun (IgM)	PRNT	Peptide ELISA Results
1	Neg	38.42	Neg	34.82	Pos	Neg	1:64	Pos
2	29.33	Neg	Neg	38.98	Neg	Neg	<1:16	Pos
3	20.21	36	38.27	25.64	Neg	Neg	<1:8	Pos
4	Neg	35.77	Neg	Neg	inconclusive	Neg	1:16	Pos
5	Neg	34.9	Neg	36.88	Pos	Pos	ND	Pos
6	Neg	29.01	Neg	36.31	Pos	Neg	1:2048	Pos
7	Neg	21.26	Neg	34.94	Pos	Neg	1:256	Pos

ND: not determined. Pos: positive result for target virus. Neg: negative for target virus.

**Table 4:** ZIKV seroprevalence among HIV-positive patients.

Sample ID	ZIKV	HIV Status *
1	Neg	Not detected
2	Neg	79
3	ND	<LOD
4	Pos	Not detected

ND = not determined. LOD = limit of detection. *—HIV genome copies per milliliter by qRT-PCR.

**Table 5:** ZIKV seroprevalence in Brazilian pediatric patients.

Sample ID	Age (Months)	IgM+	IgG+ & CT+	Peptide ELISA ZIKV
1	32	Pos	Neg	Pos
2	34	Neg	Pos	Pos
3	32	Neg	Pos	Pos
4	21	Neg	Pos	Pos
5	34	Neg	Pos	Pos
6	32	Neg	Pos	Pos
7	34	Neg	Pos	Pos
8	30	Pos	Neg	Pos
9	29	Neg	Pos	Pos
10	32	Pos	Neg	Pos
11	32	Neg	Pos	Pos
12	35	Neg	Pos	Pos
13	30	Pos	Neg	Pos
14	33	Neg	Pos	Pos
15	30	Neg	Pos	Pos
16	32	Neg	Pos	Pos
17	35	Neg	Pos	Pos
18	32	Neg	Pos	Pos
19	32	Pos	Neg	Pos
20	33	Neg	Pos	Pos
21	32	Neg	Pos	Pos
22	16	Neg	Pos	Pos

Pos: positive result for target virus. Neg: negative for target virus.

**Table 6:** Specificity and sensitivity values comparing the custom peptide-based ELISA to microneutralization assays for uncharacterized Guatemalan samples.

	ZIKV (*n*)
Sensitivity	30.19% (175)
Specificity	83.07% (175)

## Data Availability

Data is contained within the article or supplementary material.

## Supplementary Materials

The following are available online at https://www.mdpi.com/article/10.3390/v13071319/s1, Table S1: Details for all serum collections evaluated in the current study. Table S2: Sequences of virus-specific peptides incorporated into the peptide-based ELISA. Table S3: Comparative results from the Mikrogen commercial assay with the peptide-based ELISA. Table S4: Normalized results of peptide-based ELISA for all samples evaluated in the current study. Table S5: Results from peptide-based ELISA for characterized clinical sera from Sri Lanka. Table S6: Results from peptide-based ELISA for characterized clinical sera from Chile. Table S7: Comparison of various serological results to peptide-based ELISA. Table S8: Summary peptide-based ELISA results for uncharacterized sera from multiple countries. Table S9: Microneutralization results for Guatemalan samples.
